# Pleiotropic alterations in gene expression in Latin American *Fasciola hepatica* isolates with different susceptibility to drugs

**DOI:** 10.1186/s13071-017-2553-2

**Published:** 2018-01-24

**Authors:** Santiago Radio, Santiago Fontenla, Victoria Solana, Anna C. Matos Salim, Flávio Marcos Gomes Araújo, Pedro Ortiz, Cristian Hoban, Estefan Miranda, Valeria Gayo, Fabiano Sviatopolk-Mirsky Pais, Hugo Solana, Guilherme Oliveira, Pablo Smircich, José F. Tort

**Affiliations:** 1 Departamento de Genética, Facultad de Medicina, Universidad de la Republica, UDELAR, Montevideo, Uruguay; 2 Present address: Instituto de Investigaciones Biológicas Clemente 28 Estable. MEC, Montevideo 29, Uruguay; 3 Laboratorio de Biología Celular y Molecular, Facultad de Ciencias Veterinarias, Universidad Nacional del Centro de la Provincia de Buenos Aires, Tandil, Argentina; 4 Centro de Pesquisas René Rachou, Fundação Oswaldo Cruz, Belo Horizonte, Brazil; 5 Laboratorio de Inmunología, Facultad de Ciencias Veterinarias, Universidad Nacional de Cajamarca, Cajamarca, Peru; 6 Instituto Nacional de Investigaciones Forestales, Agrícolas y Pecuarias, Secretaria de Agricultura, Ganadería, Desarrollo Rural, Pesca y Alimentación, Morelos, Mexico; 7 Departamento de Parasitología, División de Laboratorios Veterinarios (DILAVE), “Miguel C. Rubino”, Ministerio de Ganadería, Agricultura y Pesca (MGAP), Montevideo, Uruguay; 8 Present address: Instituto Tecnológico Vale, Belém, Brazil; 9 Laboratorio de Interacciones Moleculares, Facultad de Ciencias, Universidad de la Republica, UDELAR, Montevideo, Uruguay

**Keywords:** *Fascola hepatica*, Drug resistance, American isolates, Triclabendazole, Albendazole, Transcriptomics

## Abstract

**Background:**

*Fasciola hepatica* is the main agent of fasciolosis, a zoonotic disease affecting livestock worldwide, and an emerging food-borne disease in humans. Even when effective treatments are available, drugs are costly and can result in tolerance, liver damage and normally they do not prevent reinfection. Drug-resistant strains in livestock have been reported in various countries and, more worryingly, drug resistance in human cases has emerged in South America. The present study aims to characterize the transcriptome of two South American resistant isolates, the Cajamarca isolate from Peru, resistant to both triclabendazole and albendazole (TCBZR/ABZR) and the Rubino isolate from Uruguay, resistant to ABZ (TCBZS/ABZR), and compare them to a sensitive strain (Cenapa, Mexico, TCBZS/ABZS) to reveal putative molecular mechanisms leading to drug resistance.

**Results:**

We observed a major reduction in transcription in the Cajamarca TCBZR/ABZR isolate in comparison to the other isolates. While most of the differentially expressed genes are still unannotated, several trends could be detected. Specific reduction in the expression levels of cytoskeleton proteins was consistent with a role of tubulins as putative targets of triclabendazole (TCBZ). A marked reduction of adenylate cyclase might be underlying pleiotropic effects on diverse metabolic pathways of the parasite. Upregulation of GST mu isoforms suggests this detoxifying mechanism as one of the strategies associated with resistance.

**Conclusions:**

Our results stress the value of transcriptomic approaches as a means of providing novel insights to advance the understanding of drug mode of action and drug resistance. The results provide evidence for pleiotropic variations in drug-resistant isolates consistent with early observations of TCBZ and ABZ effects and recent proteomic findings.

**Electronic supplementary material:**

The online version of this article (10.1186/s13071-017-2553-2) contains supplementary material, which is available to authorized users.

## Background

Fasciolosis is indisputably one of the most widely distributed zoonotic diseases, affecting no less than 300 million cattle and 250 million sheep worldwide. The economical cost of the disease has been valued at 3 billion dollars annually [1, 2]. This huge economic impact from direct losses might be an underestimate considering indirect costs of treatment, or loss of animal workforce in less industrialized countries. Furthermore, fasciolosis is emerging as a relevant issue in human health, affecting roughly 2.6 million people worldwide. For this reason it has been considered as a re-emerging neglected disease by the WHO [3]. In the Americas the disease is widespread in livestock and is an important human food-borne infection in the Altiplano region of Bolivia and Peru [4].

Although effective treatments are available, drugs are costly and usually do not block reinfection. Liver fluke drug resistance is a preoccupying productive problem in Latin America and a concern in medicine since human cases have emerged [4]. Triclabendazole (TCBZ) treatment failure has been reported in Brazil [5], Argentina [6] and Peru [7–9]. Moreover, resistance to albendazole (ABZ) has been reported in Argentina [10], Uruguay [11], Chile and Bolivia [5]. More worryingly, ABZ resistance accompanied by reduced effectivity of TCBZ has been registered in Bolivia [12] and Peru [13], and resistance to both drugs has been described in an isolate from the Cajamarca valley of Peru [9, 11]. This phenomenon of double resistance compromises the idea of combined drug treatments. More recently, TCBZ resistance in humans has been reported in Chile and Peru [14, 15]. There is a pressing need to understand epidemiological and mechanistic aspects of drug resistance emergence.

Despite being in use for more than 30 years, the exact mechanism of action of TCBZ is still not completely elucidated [16]. One of the earliest and more complete biochemical studies provides early evidence of pleiotropic effects [17]. The study documented that the drug was absorbed through the tegument affecting the motility of the parasite in a dose-dependent manner. This effect was associated with changes in the worm’s resting tegument membrane potentials. In addition, an effect on tubulin binding was also observed. Furthermore, an anaerobic metabolic stimulation was suggested by the increase in propionate and acetate production without affecting ATP levels. A general reduction of secreted proteases was also described [17], an effect that might be associated with a reported general inhibition of protein synthesis [18]. Several studies have focused on tegument disruption, one of the major effects of drug treatment [19–24]. This was generally associated with a putative role of tubulin as target, based on similarities to findings on other benzimidazole effects against nematodes [16, 25]. The ability to compete with colchicine, a known inhibitor of tubulin polymerization, was an early test for tubulin binding [17, 26]. The role of microtubules in TCBZ pathogenesis was further suggested by several lines of evidence, like the detection of cell division inhibition in vitelline and reproductive cells [27–29], the reduction in the transport of tegumental secretory bodies [22], and the inhibition of tubulin immunostaining [30, 31]. Surprisingly, TCBZ was recently reported to inhibit adenylate cyclase activity in yeast, activating the stress response [32]. This effect has not been observed in the liver fluke so far, but considering the role of cAMP as second messenger, it might provide an explanation for the pleiotropic effects of the drug.

Interestingly, TCBZ is quite specific for fasciolids, being ineffective against nematodes. On the other hand, out of the benzimidazolic drugs used for gastrointestinal roundworms, only ABZ is effective against the adult stage of *Fasciola* spp. [33]. These differences and the fact that ABZ is usually effective against TCBZ-resistant isolates suggest that different mechanisms might be underlying the effect of each benzimidazolic drug. ABZ also induces tegument damage, disruption of tegumental vesicle traffic, alterations in reproductive tissues and vitelline cells, and reduction in egg production [33, 34]. However, despite these similarities, differences in the metabolism of worms treated with these drugs are suggestive of diverse targets or mechanisms.

It has been shown that the metabolism of TCBZ to triclabendazole sulphoxide (TCBZ.SO) and TCBZ.SO to triclabendazole sulphone (TCBZ.SO2) is greater in TCBZ-R than in TCBZ-S isolates [35–37]. Interestingly, the uptake of TCBZ and TCBZ.SO by TCBZ-R fluke isolates is significantly lower than in TCBZ-S flukes, while the uptake of ABZ is similar in both strains [35, 38]. The effect can be reversed by incubating the TCBZ-R flukes in the presence of ivermectin, a substrate of P-glycoprotein (PGP) drug efflux pump. While disruption of the tegument is markedly reduced in TCBZ-R flukes, the co-incubation with R(+)-verapamil, another PGP inhibitor, gives rise to severe tegumental lesions [39, 40], highlighting PGP as one of the possible detoxifying mechanisms. A similar effect of increased tegument disruption is seen when TCBZ-R flukes are incubated with methimazole, an inhibitor of the flavin mono-oxigenases (FMO) [41]. Ketoconazole, an inhibitor of CYP450 [42, 43] also produce as similar phenotype, suggesting that these pathways might be associated with drug resistance as well. It was hypothesized that some of these enzymes might be upregulated in the resistant strains [35, 36]. Consistent with this, an increased enzyme activity of detoxifying enzymes gluthatione S-transferase (GST), carboxyl esterase and carbonyl reductase, was observed in TCBZ-treated worms [44], and a higher GST response was observed in the TCBZ-R Sligo strain in comparison to the Cullompton sensitive isolate [45]. A comparative proteomic study of the Sligo and Cullompton isolates showed variation in energy metabolic enzymes, detoxifying enzymes and structural proteins, confirming the pleiotropic nature of TCBZ effects, and reinforced suggestions of differential expression of several proteins [46].

It is therefore clear that anthelmintic drugs produce complex effects on the liver fluke, affecting the metabolism, physiology and morphology of the parasite. Drug-resistant isolates seem to utilize diverse detoxifying mechanisms to cope with these drugs [47]. We sought to shed new light into drug resistance by using a genomics approach, which has been demonstrated to be very powerful in schistosomes [48–52]. Similar efforts have been initiated in *F. hepatica* [53], starting from well characterized European TCBZ-R and TCBZ-S isolates and their genome sequences [54, 55]. In this work we add a transcriptomic perspective to the current knowledge via an in depth analysis of the basal transcriptomic state of three isolates from the Americas with different susceptibilities to TCBZ and ABZ. This report would set the baseline for upcoming comparative studies on variations of gene expression upon exposure to the different anthelminthic drugs.

## Methods

### Strains

Three *Fasciola hepatica* isolates with different susceptibilities to triclabendazole (TCBZ) and albendazole (ABZ) were analyzed. The “Cajamarca” isolate was originally obtained by Dr Pedro Ortiz from infected cattle in Cajamarca, Peru. It has been maintained for five years in sheep and characterized in their laboratory as resistant to both ABZ and TCBZ [9, 11]. The “Rubino” isolate (originally obtained from cattle in Salto, Uruguay) is resistant to ABZ but sensitive to TCBZ [11]. It has been maintained in sheep for eight years by Dr Valeria Gayo in the DILAVE “Miguel C. Rubino”. The isolate is routinely used to test formulations of Closantel and TCBZ, since it is sensitive to both. Similarly, “Cenapa” is an isolate sensitive to both drugs that has been maintained for more than a decade in sheep, and is routinely used by the veterinary health authorities of the Mexican government to evaluate the efficacy of anthelminthics. The Cenapa isolate was kindly provided by Dr Estefan Miranda. The three laboratories followed protocols approved by the respective local Committees of Animal Experimentation, in accordance to the recommendations of Guide for the Care and Use of Laboratory Animals [56]. All isolates are maintained in sheep without selective drug pressure.

### RNA-sequencing and pre-processing of the reads

Adult flukes were obtained from infected sheep livers and stored immediately in RNAlater. No macroscopic morphological differences were observed between flukes. PolyA+ RNA was purified from single adult worms of the different isolates in duplicates and used to generate paired end (PE) libraries using the TrueSeq LT kit (Illumina, San Diego, USA). Samples were sequenced in the Illumina Platform at the CPqRR sequencing facilities at FIOCRUZ, Belo Horizonte, to obtain 117 million 76 bp pair end reads. The resulting sequences were quality trimmed and mapped to the *F. hepatica* reference genome (WormBase Parasite Acc: PRJEB6687) [54] using CLC Genomics Workbench v7 (Qiagen, Aarhus C, Denmark) with default parameters. A good coverage of predicted genes (over 80%) was observed. A summary of the number of reads obtained in each step is shown in Additional file 1: Table S1. Raw sequencing data were submitted to SRA under accession PRJNA339158.

### Differential expression (DE) determination

Differential expression was analyzed using different tools of the Bioconductor suite of bioinformatics packages [57, 58]. To obtain expression estimates, mapped reads were counted for each gene using the summarizeOverlaps function from the GenomicAlingments package [59] and log_2_-transformed. To account for sequencing depth in differential expression analysis, raw read counts were normalized with DESeq2 [60]. Replicate consistency was established by computing pairwise Pearson’s correlation coefficients in R. Differentially expressed genes were defined using DESeq2 (using the Wald Test implemented in the package with 4 degrees of freedom) from pairwise comparisons of the log_2_-transformed normalized expression estimates, establishing a minimum fold change of 2 and a false discovery rate (FDR) (controlled using the Benjamini & Hochberg’s method [61]) corrected *P*-value lower than 0.05.

### Functional enrichment analysis

Lists of DE genes were analyzed with the TopGO Biocondutor package in R [62] to assess enriched gene ontology categories (using Fisher’s exact test implemented in the package with 2 degrees of freedom; a *P*-value < 0.01 was considered significant). The parameters orderBy = “classicFisher” and ranksOf = “classicFisher” were set for visualization. GO annotation was retrieved from Wormbase Parasite (parasite.wormbase.org) [63].

### SNP calling

SNPs were called from RNAseq mappings to the reference genome using mpileup (-uf parameters) and bcftools (-mv parameters) from samtools [64]. Putative phenotypic effects of the variants called were assessed with the variant effect predictor script (part of the Ensembl tools [65]. Synonymous codon variants and other low impact mutations were not included in the analysis.

### KEGG orthology annotation

The GhostKOALA tool [66] was used to annotate the predicted proteome. Genes belonging to common housekeeping functions were identified using the KEGG Brite reconstruction list [67]. Selected categories, gene IDs and genome annotation are shown in Additional files as indicated in the text.

## Results and discussion

### A lower transcription level is observed for a significant amount of Cajamarca genes

**Fig. 1:**
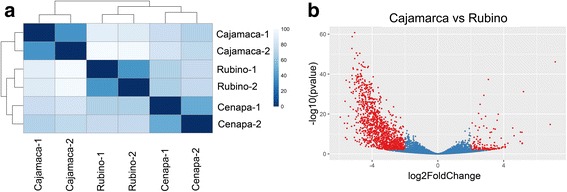
Overview of sample distances and differential expression. **a** Heatmap of the Euclidean sample to sample distances shown in a white to blue scale (blue represents higher correlation). Both replicates for each sample are shown, with the corresponding clustering dendrograms on the sides. **b** Volcano plot showing the differential expression between the Cajamarca and Rubino transcripts. Red dots represent differentially expressed genes (log2 fold change > 2, *P*-value < 0.01)

To obtain a global picture of the differences of the transcriptomic profiles of the different isolates, samples were clustered and pairwise correlations were computed. Figure 1a shows that while duplicates were very consistent, the Cajamarca isolate displayed the lowest level of correlation to the other samples. This difference is mostly explained by comparatively low mRNA steady state levels of many transcripts in this strain (Fig. 1b and Additional file 2: Figure S1a). On the other hand, differences in the gene expression were subtler between the Rubino and Cenapa samples (Additional file 2: Figure S1b).

### Housekeeping gene expression is not biased between isolates

**Fig. 2:**
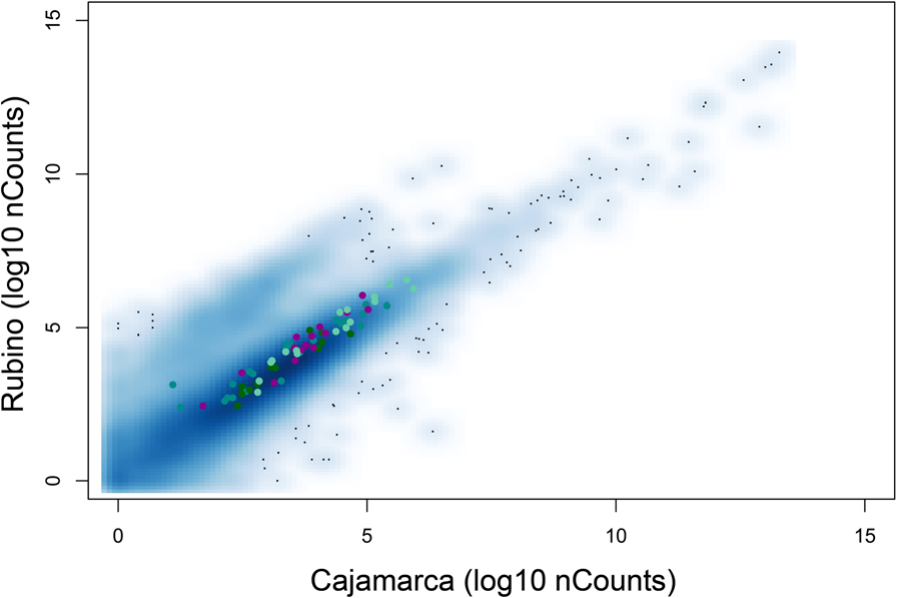
Correlation of housekeeping gene expression. Scatterplot showing the correlation of Rubino normalized counts per gene *versus* their Cajamarca counterparts. Filled circles highlight the expression of genes belonging to housekeeping functions: dark cyan, aminoacyl tRNA synthetases; dark green, core DNA polymerase subunits; dark magenta, core proteasome subunits; light green, core spliceosome subunits

Since we saw a reduction of transcription in the TCBZR/ABZR Cajamarca isolate in relation to the other two isolates, we wanted to check if the asymmetrical distribution of differentially expressed genes (DEG) among the strains was due to a general lower transcript level in the Cajamarca isolate that normalization was not able to account for. Interestingly, we detected that housekeeping genes (such as aminoacyl tRNA synthetases, DNA polymerase subunits, proteasome subunits and spliceosome subunits) do not show differences of expression among any of the strains (Fig. 2 and Additional file 3: Figure S2). These results indicate that Cajamarca samples are not globally skewed toward less expression, but rather different gene families might be differentially affected. These results are puzzling since no drug selective pressure was applied to these worms, and it might reflect a basal status of the isolate. Notably, early studies of the effect of TCBZ reported a marked drop in protein synthesis [18], consistent with morphological observations of changes in heterochromatin, the disappearance of the nucleolus and the subsequent reduction of ribosomes, reduction in Golgi complexes, and secretory bodies in tegumental cells [22]. Therefore, it is tempting to consider that the study of differentially expressed (DE) genes might help to pinpoint some possible candidates involved in the resistance phenotype.

### The top differentially expressed genes are mostly not annotated

Pairwise comparisons were performed between the isolates using DESeq2 package. Almost half of the downregulated or upregulated genes in each pairwise list are currently devoid of any annotation. We focused on the top 20 upregulated or downregulated DEG in each pairwise comparison (Additional file 4: Table S2). Even within this selected set, 41 out of 58 of the downregulated DEG are of unknown function, and diverse functions are present in the remaining annotated genes. A similar scenario was observed for the upregulated DEG where 37 of 47 were unannotated, highlighting the still incomplete nature of the available genome annotation. However, 3 out of 10 of the annotated DEGs upregulated in the Cajamarca and Rubino resistant strains, contain putative CUB domains (IPR000859). This domain (for Complement C1r/C1s, Uegf and Bmp1) comprises more than 100 amino acids and is usually found in the extracellular- or plasma membrane-associated proteins with diverse functions. Several mammalian CUB containing proteins are proteases with calcium binding EGF domains, taking part in pleiotropic functions like complement activation, developmental patterning, neurotransmission and cell signaling [68]. CUB domain containing proteins found in the *F. hepatica* genome are generally short with no other associated domains, but this might well be a consequence of the still fragmented nature of the assembly. Interestingly, upregulation of CUB domain containing proteins has been observed in *C. elegans* in response to albendazole administration [69]. Although little is known of the relevance or function of these proteins in helminths, the upregulation in the resistant isolates even without exposure to the drug is noteworthy.

### Cytoskeleton related genes are less expressed in the Cajamarca strain

**Fig. 3:**
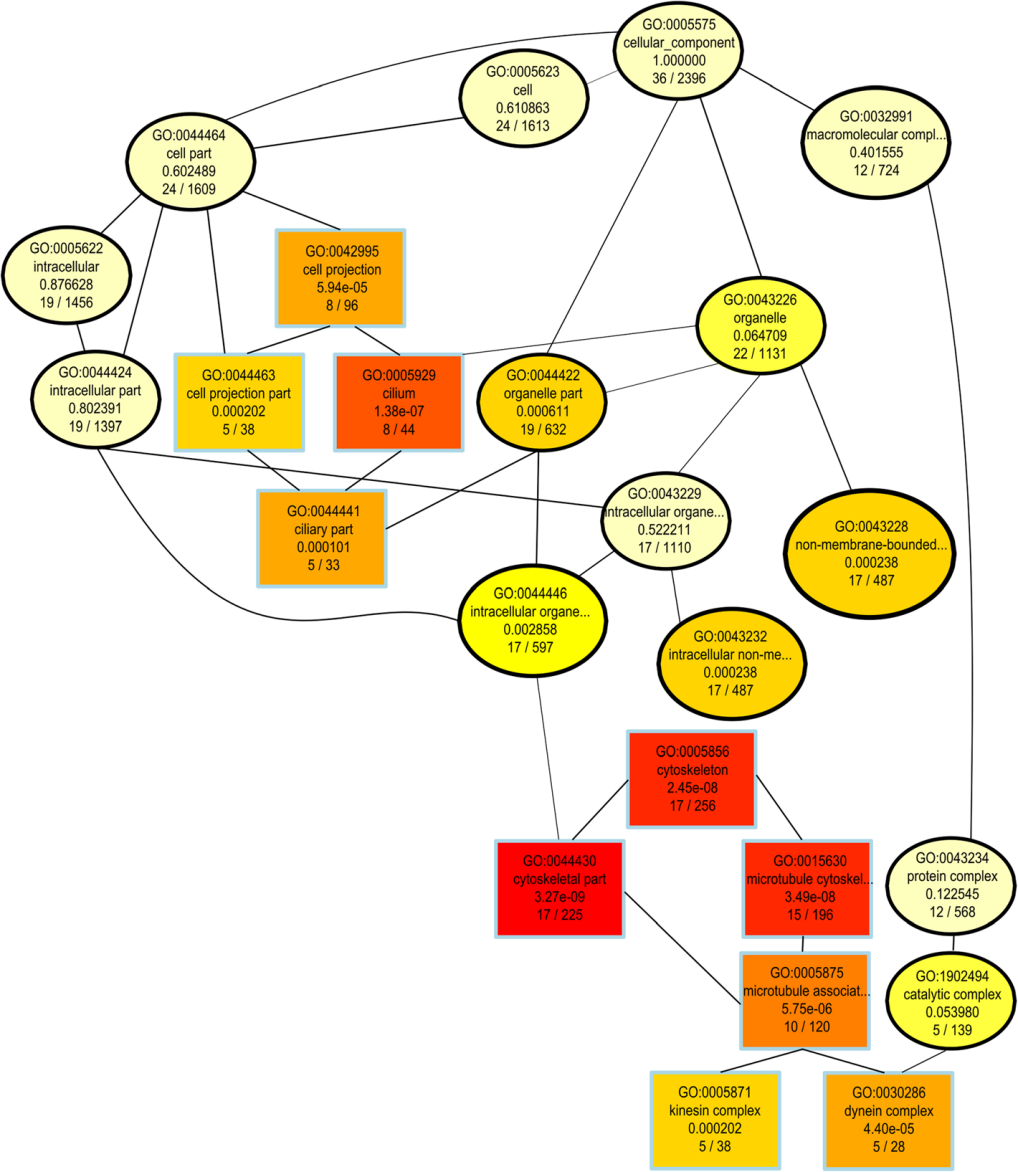
Gene ontology (GO) enrichment of Cajamarca downregulated genes. Directed acyclic graph showing cellular component GO categories present in the downregulated genes when comparing the Cajamarca with the Cenapa samples. Colors indicate category overrepresentation, with red being most significant. Text lines indicate GO number, category description, Fisher’s *P*-value and the number of genes in the set over the total number of genes in the genome for the GO category

The entire lists of DEG in each pairwise comparison were subjected to GO functional category enrichment analysis. Remarkably, several terms related to cytoskeleton structure and function showed a significant enrichment in the list of downregulated genes in the resistant strains, especially in the Cajamarca isolate (Fig. 3 and Additional file 5: Table S3). This is an interesting observation taking into account the putative role of tubulins as targets of ABZ, TCBZ and other benzimidazole-based drugs [70, 71].

**Fig. 4:**
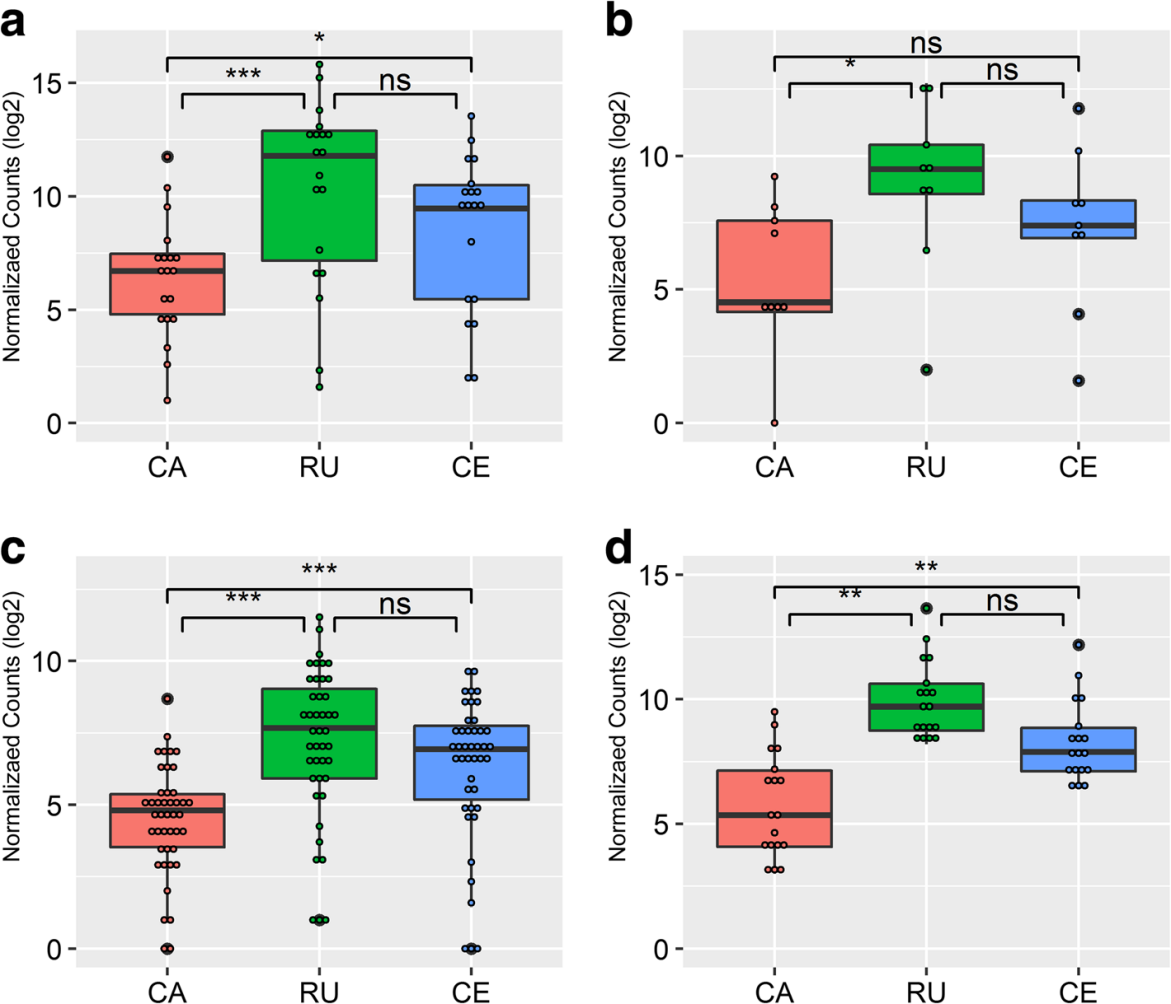
Expression levels of cytoskeleton related gene families. Boxplot showing the expression levels for mRNAs coding for α-tubulins (**a**), β-tubulins (**b**), kinesins (**c**) and dyneins (**d**) in each sample. Samples were compared using a two-sided Student’s *t*-test in R. For α-tubulins CA *vs* RU: *t*_(30)_ = -3.586, *P* < 0.001; CA *vs* CE: *t*_(33)_ = -2.148, *P* = 0.039; RU *vs* CE: *t*_(35)_ = 1.515, *P* = 0.139. For β-tubulins CA *vs* RU: *t*_(15)_ = -2.529, *P* = 0.023; CA *vs* CE: *t*_(16)_ = -1.372, *P* = 0.189; RU *vs* CE: *t*_(16)_ = 1.162, *P* = 0.262. For kinesins CA *vs* RU: *t*_(64)_ = -5.092, *P* < 0.001; CA *vs* CE: *t*_(67)_ = -3.522, *P* < 0.001; RU *vs* CE: *t*_(73)_ = 1.608, *P* = 0.112. For dyneins CA *vs* RU: *t*_(347)_ = -2.473, *P* = 0.014; CA *vs* CE: *t*_(343)_ = -0.207, *P* = 0.836; RU *vs* CE: *t*_(337)_ = 2.407, *P* = 0.017. Significant comparisons are marked with asterisks (*P ≤ 0.05, **P ≤ 0.01, ***P ≤ 0.001). *Abbreviations*: CA, Cajamarca; RU, Rubino; CE, Cenapa; ns, not significant

These results prompted us to further characterize the expression of cytoskeleton-related gene families. Figure 4 shows the expression profile of the α and β tubulin gene families (Fig. 4a, b) and motor protein families (kinesins and dyneins Fig. 4c, d). In all cases the Cajamarca isolate showed lower levels of gene expression for these families when compared to the other strains, which were not significantly different among them (Student’s *t*-test *P* < 0.05, see legend of Fig. 4 for exact *P*-values of each comparison). In particular, α-tubulin and β-tubulin mRNAs showed differential expression between the strains being down-represented in the Cajamarca isolate (Fig. 4a, b and Additional file 6: Table S4). Finding a skewed expression of tubulins and other cytoskeleton genes in a double resistant isolate reinforces the notion of tubulin as a putative TCBZ target.

SNPs in the β-tubulin genes have been reported as molecular mechanisms of resistance development in nematodes, and in particular three amino acid substitutions in the *Haemonchus contortus* β-tubulin have been associated with resistance [72, 73]. Initial studies attempting to find the same variations in *F. hepatica* failed to find association between these changes and resistant status [30, 74]. Despite this, a strongly reduced tegument damage and little disruption of tubulin immunostaining were observed in TCBZ resistant flukes in comparison to a sensitive isolate [30]. Similarly, disruption of tegument and reduction of tubulin immunostaining were also observed upon exposure to albendazole sulphoxide (ABZ-SO) [34] highlighting tubulin as one of the putative targets in *F. hepatica*. Our results support the association of these proteins with the resistance phenomenon, but in our case, mRNA level variations and not SNPs were revealed as the putative molecular mechanism. Notably, a similar reduction in tubulin expression has been observed in *H. contortus* drug-resistant strains [75, 76]. However, a previous study failed to detect differences in transcription levels of diverse β-tubulins between the TCBZ resistant Oberon and the Leon TCBZ sensitive isolates [77]. While these contradictory observations might be pointing to different mechanisms of resistance in different strains, the observation that other motor proteins mRNA levels are reduced (Fig. 4c, d) clearly relates to the previous finding and further associates the resistance phenotype with microtubule cytoskeleton function.

Interestingly, collagen coding mRNAs were under-expressed in the Rubino isolate compared to the other strains. The difference is particularly significant with the Cajamarca isolate where an average 5-fold transcription level was detected for collagen (Additional file 7: Table S5).

### Only a few genes associate with detoxifying pathways are differentially expressed

Since several putative candidates genes involved in drug resistance have been advanced [47, 78], we verified their differential expression in our three isolates. One of the first mechanisms proposed in detoxification is the oxidation of the incoming drug by proteins involved in redox activity (defined by annotation of functional domains) (Additional file 8: Table S6). We detected downregulation of several redox proteins in the Cajamarca strain when compared with the other two isolates, and we observed extreme variations.

Another proposed mechanism for drug resistance is the conjugation of the primary metabolite to proteins, such as the glutathione S-transferase family (GST), and other detoxification enzymes. The expression pattern of these candidates was analyzed, and no significant differences were observed for the complete set when a FC > 2 cut-off was applied. However, while there is no global trend when all enzymes are considered, it is interesting to point out that some GST genes, particularly of the mu type, do have statistically significant variation and a modest upregulation in the resistant strains (FC > 2, see Additional file 9: Table S7). This is consistent with previous biochemical studies that have shown increased GST activity in the Sligo resistant strain when compared to the Cullampton sensitive strain [44, 45]. Also an increase in GST mu was observed in the only comparative proteomic study available so far [46].

Among the detoxification proteins, some mRNAs were significantly different between the strains (Additional file 9: Table S7). It has been hypothesized that ABC transporters might be upregulated in drug-resistant strains [79]. Indeed, an ABC transporter-like protein (BN1106_s3396B000087) was upregulated in the TCBZ resistant isolate (Additional file 9: Table S7). Whether this is further upregulated upon drug exposure still needs to be addressed.

### Adenylate cyclase is reduced in TCBZR isolate

Recently it was reported that TCBZ can inhibit adenylate cyclase (AC) activity in yeast [32], but despite being widely studied in *F. hepatica* [80], there are still no in vitro studies of AC variation in response to drug administration. To gain insight into this possible mechanism, we investigated if AC expression was skewed in our samples. Surprisingly we observed a significant variation on the expression levels of several AC isoforms in both the resistant isolates in relation to the Cenapa sensitive strain. Notably, all isoforms tend to have consistently low transcription levels in the Cajamarca isolate with some of them being significant, while variations in both directions were found in the ABZR Rubino isolate (Table 1).

**Table 1:** Pairwise comparisons for all annotated adenylate cyclase genes

Gene	Cajamarca *vs* Cenapa		Rubino *vs* Cenapa		Cajamarca *vs* Rubino		Genome annotation	Kegg annotation
	Fold change (log2)	FDR	Fold change (log2)	FDR	Fold change (log2)	FDR		
BN1106_s820B000180	**-2.54**	1.00e-06	0.78	1.49e-01	**-3.32**	1.42e-11	Adenylyl cyclase class-3/4/guanylyl cyclase (Nucleotide cyclase)	K08049 ADCY9
BN1106_s5842B000024	**-2.42**	4.10e-03	*1.78*	3.80e-03	**-4.20**	7.78e-09	Adenylyl cyclase class-3/4/guanylyl cyclase (Nucleotide cyclase)	K08049 ADCY9
BN1106_s1582B000143	**-1.81**	1.26e-04	*1.24*	5.50e-03	**-3.05**	9.42e-13	Adenylyl cyclase class-3/4/guanylyl cyclase (Nucleotide cyclase)	–
BN1106_s3088B000130	**-1.74**	3.43e-02	*1.36*	5.27e-02	**-3.10**	6.20e-06	Adenylyl cyclase class-3/4/guanylyl cyclase (Nucleotide cyclase)	–
BN1106_s2758B000091	**-1.61**	1.20e-02	*1.67*	2.09e-03	**-3.28**	8.90e-10	Adenylyl cyclase class-3/4/guanylyl cyclase (Nucleotide cyclase)	K08041 ADCY1
BN1106_s795B000308	-1.05	7.29e-02	**-1.91**	2.65e-04	0.85	2.40e-01	Adenylyl cyclase class-3/4/guanylyl cyclase (Nucleotide cyclase)	K08049 ADCY9
BN1106_s451B000364	-0.58	6.54e-01	**-1.98**	2.50e-02	1.40	2.07e-01	Adenylyl cyclase class-3/4/guanylyl cyclase (Nucleotide cyclase)	K08049 ADCY9
BN1106_s4307B000027	-0.48	na	-0.61	na	0.00	na	na	K08049 ADCY9
BN1106_s1588B000215	-0.12	na	-1.02	na	0.91	6.46e-01	Adenylyl cyclase class-3/4/guanylyl cyclase (Nucleotide cyclase)	K08049 ADCY9
BN1106_s1588B000216	0.13	9.35e-01	-0.78	4.20e-01	0.91	4.60e-01	Adenylyl cyclase class-3/4/guanylyl cyclase (Nucleotide cyclase)	K08049 ADCY9

IDs of DEG genes [FC > 2 or < -2 (log2 FC >1 or < -1) and an FDR < 0.05] in any comparison are highlighted in bold. The log2FC value is highlighted in italics and underlined for the upregulated genes and in bold and double underlined for the downregulated ones

The described inhibition of AC upon exposure to TCBZ in yeast would not necessarily reduce their transcription levels. Considering the central role of AC in metabolism, its downregulation in the Cajamarca isolate may account for the pleiotropic reduction in gene expression observed. Since cyclic AMP is a relevant second messenger, we investigated the expression levels of the main mediators Protein Kinase A (PKA) and the RAP guanine nucleotide exchange factors. Both genes showed no alterations in their transcription profile. Nevertheless, the reduction in AC might result in a concomitant AMPc drop, which in turn would activate diverse stress responses through all these effectors without altering their transcription levels. This might provide an explanation for the pleiotropic effects on motility, cytoskeleton, carbohydrate metabolism, and activation of stress and detoxifying enzymes. In any case, carefully controlled in vitro experiments of TCBZ inhibition of AC activity in flukes are needed to confirm these hypotheses.

Interestingly, our results are generally consistent with the comparative proteomics results that found variations in several metabolic enzymes, as well as in stress response proteins and structural proteins [46]. Several of the proteins and genes found to be differentially expressed at transcript level in this study (Additional file 10: Table S8) were also reported as differential in the previous proteomics work, such as the detoxifying enzymes already mentioned (redox proteins and GSTs).

## Conclusions

In the first transcriptomic analysis of *F. hepatica* isolates with different levels of drug susceptibility, we were able to highlight diverse protein functions and families that show differential gene expression. Notably, several of the affected genes and pathways correspond to those that are being proposed as normally altered upon drug exposure. The presence of variation in expression levels in these pathways in resistant isolates is suggestive, but we cannot conclude with the available evidence that it is related to the resistant phenotype. Further experiments assessing expression levels of these mRNAs in the different isolates upon controlled exposure are necessary to either confirm or reject that RNA levels actually vary upon drug exposure. While those experiments are on the way, we can highlight that the differentially expressed genes in resistant isolates are diverse, and correlate quite well with initial biochemical and structural observations of the pleiotropic nature of drug effects, particularly in the case of TCBZ [17–24]. Moreover they also are coincident with more recent proteomic characterization of drug sensitive and resistant isolates from European origins [46]. Interestingly a recent transcriptomic study of drug-resistant isolates of *Trypanosoma cruzi* also showed altered expression of genes associated with putative drug action mechanisms [81]. The fact that the parasites can survive drug exposure strongly suggests that resistance is the result of additive subtle changes in the expression, and consequently protein metabolic activity. A corollary of this observation is that resistance in different isolates might rely on diverse mechanisms or targets. This highlights the need for studying diverse isolates in order to gain a better understanding of drug action and parasite resistance mechanisms. The results provided by this work are a step in this direction that we hope will impact future methods for parasite control.

## Additional files

Additional file 1: Table S1. General data processing overview. (XLSX 10 kb)

Additional file 2: Figure S1. Differential expression between isolate pairs. Volcano plot showing the differential expression of transcripts between Cajamarca and Cenapa (**a**) and between Rubino and Cenapa (**b**). Red dots represent differentially expressed genes (log2 fold change > 2, *P*-value < 0.01). (TIFF 1086 kb)

Additional file 3: Figure S2. **a** Correlation of normalized counts and housekeeping genes. Scatterplot showing the correlation of Cenapa normalized counts per gene *versus* their Cajamarca counterparts. **b** Scatterplot showing the correlation of Cenapa normalized counts per gene *versus* their Rubino counterparts. Full circles highlight the expression of genes belonging to housekeeping functions. Colors are as in Fig. 2. (TIFF 1042 kb)

Additional file 4: Table S2. Top 20 upregulated (**a**) and downregulated (**b**) genes for each pairwise comparison. (XLSX 16 kb)

Additional file 5: Table S3. GO category overrepresentation analysis for each two-way comparison. (XLSX 23 kb)

Additional file 6: Table S4. Differential expression of cytoskeleton related gene families. (XLSX 23 kb)

Additional file 7: Table S5. Differential expression of collagen genes. (XLSX 51 kb)

Additional file 8: Table S6. Differential expression of redox genes. (XLSX 40 kb)

Additional file 9: Table S7. Differential expression of detoxifying enzymes. (XLSX 3441 kb)

Additional file 10: Table S8. Differentially expression of genes identified in proteomic study. (XLSX 19 kb)

## Abbreviations

ABZ: Albendazole
ABZR: Albendazole-resistant
ABZS: Albendazole-sensitive
ABZ-SO: Albendazole sulphoxide
AC: Adenylate cyclase
DEG: Differentially expressed genes
FDR: False discovery rate
FMO: Flavin mono-oxigenases
GST: Gluthatione S-transferase
PGP: P-glycoprotein
PKA: Protein kinase A
TCBZ: Triclabendazole
TCBZ.SO: Triclabendazole sulphoxide
TCBZ.SO2: Triclabendazole sulphone
TCBZR: Triclabendazole-resistant
TCBZS: Triclabendazole-sensitive

## References

[1] Keiser J, Utzinger J (2009). Food-borne trematodiases. Clin Microbiol Rev.

[2] Charlier J, Vercruysse J, Morgan E, van Dijk J, Williams DJL (2014). Recent advances in the diagnosis, impact on production and prediction of *Fasciola hepatica* in cattle. Parasitology.

[3] Fürst T, Duthaler U, Sripa B, Utzinger J, Keiser J (2012). Trematode infections: liver and lung flukes. Infect Dis Clin N Am.

[4] Carmona C, Tort JF (2017). Fasciolosis in South America: epidemiology and control challenges. J Helminthol.

[5] Oliveira DR, Ferreira DM, Stival CC, Romero F, Cavagnolli F, Kloss A (2008). Triclabendazole resistance involving *Fasciola hepatica* in sheep and goats during an outbreak in Almirante Tamandare, Paraná, Brazil. Rev Bras Parasitol Vet.

[6] Olaechea F, Lovera V, Larroza M, Raffo F, Cabrera R. Resistance of *Fasciola hepatica* against triclabendazole in cattle in Patagonia (Argentina). Vet Parasitol. 2011;178:364–6. 10.1016/j.vetpar.2010.12.047 21277090

[7] Espinoza JR, Terashima A, Herrera-Velit P, Marcos LA (2010). Human and animal fascioliasis in Peru: impact in the economy of endemic zones. Rev Peru Med Exp Salud Publica.

[8] de Dios Rojas J (2012). Resistance of *Fasciola hepatica* to triclabendazole in cattle of the Cajamarca countryside. Rev Vet Argentina.

[9] Ortiz P, Scarcella S, Cerna C, Rosales C, Cabrera M, Guzmán M, et al. Resistance of *Fasciola hepatica* against Triclabendazole in cattle in Cajamarca (Peru): a clinical trial and an in vivo efficacy test in sheep. Vet Parasitol. 2013;195(1-2):118-21. 10.1016/j.vetpar.2013.01.001 23352107

[10] Sanabria R, Ceballos L, Moreno L, Romero J, Lanusse C, Alvarez L (2013). Identification of a field isolate of *Fasciola hepatica* resistant to albendazole and susceptible to triclabendazole. Vet Parasitol.

[11] Canevari J, Ceballos L, Sanabria R, Romero J, Olaechea F, Ortiz P (2014). Testing albendazole resistance in *Fasciola hepatica*: validation of an egg hatch test with isolates from South America and the United Kingdom. J Helminthol.

[12] Mamani W, Condori R (2009). Anthelminthic resistance (*Fasciola hepatica*) in sheep against albendazole and triclabendazole, La Paz - Bolivia. Rev Inv Vet Peru.

[13] Chavez A, Sanchez L, Arana C, Suarez F (2012). Resistance to anthelmintics and prevalence of bovine fasciolosis in dairy farms in Jauja. Peru Rev Inv Vet Peru.

[14] Gil LC, Díaz A, Rueda C, Martínez C, Castillo D, Apt W (2014). Resistant human fasciolasis: report of four patients. Rev Med Chil.

[15] Cabada MM, Castellanos-Gonzalez A, Lopez M, Caravedo MA, Arque E, White AC (2016). *Fasciola hepatica* infection in an indigenous community of the Peruvian jungle. Am J Trop Med Hyg.

[16] Brennan GP, Fairweather I, Trudgett A, Hoey E, McCoy, McConville M (2007). Understanding triclabendazole resistance. Exp Mol Pathol.

[17] Bennett JL, Kohler P (1987). *Fasciola hepatica*: action *in vitro* of triclabendazole on immature and adult stages. Exp Parasitol.

[18] Stitt AW, Fairweather I, Mackender RO (1995). The effect of triclabendazole (“Fasinex”) on protein synthesis by the liver fluke, *Fasciola hepatica*. Int J Parasitol.

[19] Halferty L, Brennan GP, Hanna REB, Edgar HW, Meaney MM, McConville M (2008). Tegumental surface changes in juvenile *Fasciola hepatica* in response to treatment in vivo with triclabendazole. Vet Parasitol.

[20] Toner E, Brennan GP, Hanna REB, Edgar HW, Fairweather I (2010). Tegumental surface changes in adult *Fasciola hepatica* in response to treatment in vivo with triclabendazole in the sheep host. Vet Parasitol.

[21] Shareef PAA, Brennan GP, McVeigh P, Khan MAH, Morphew RM, Mousley A (2014). Time-dependent tegumental surface changes in juvenile *Fasciola gigantica* in response to triclabendazole treatment in goat. Acta Trop.

[22] Stitt AW, Fairweather I (1994). The effect of the sulphoxide metabolite of triclabendazole (‘Fasinex’) on the tegument of mature and immature stages of the liver fluke, *Fasciola hepatica*. Parasitology.

[23] Stitt AW, Fairweather I (1993). *Fasciola hepatica*: tegumental surface changes in adult and juvenile flukes following treatment in vitro with the sulphoxide metabolite of triclabendazole (Fasinex). Parasitol Res.

[24] Toner E, Brennan GP, Hanna REB, Edgar HW, Fairweather I (2010). Time-dependent changes to the tegumental system and gastrodermis of adult *Fasciola hepatica* following treatment *in vivo* with triclabendazole in the sheep host. Vet Parasitol.

[25] Hanna R (2015). *Fasciola hepatica*: histology of the reproductive organs and differential effects of triclabendazole on drug-sensitive and drug-resistant fluke isolates and on flukes from selected field cases. Pathogens.

[26] Fetterer RH (1986). The effect of albendazole and triclabendazole on colchicine binding in the liver fluke *Fasciola hepatica*. J Vet Pharmacol Ther.

[27] Hanna REB, Edgar HWJ, McConnell S, Toner E, McConville M, Brennan GP (2010). *Fasciola hepatica*: histological changes in the reproductive structures of triclabendazole (TCBZ)-sensitive and TCBZ-resistant flukes after treatment *in vivo* with TCBZ and the related benzimidazole derivative, compound alpha. Vet Parasitol.

[28] Stitt AW, Fairweather I (1996). *Fasciola hepatica*: disruption of the vitelline cells in vitro by the sulphoxide metabolite of triclabendazole. Parasitol Res.

[29] Stitt AW, Fairweather I (1992). Spermatogenesis in *Fasciola hepatica*: an ultrastructural comparison of the anthelmintic, triclabendazole (“Fasinex”) and the microtubule inhibitor, tubulozole. Invertebr Reprod Dev.

[30] Robinson M, Trudgett A, Hoey EM, Fairweather I (2002). Triclabendazole-resistant *Fasciola hepatica*: β-tubulin and response to in vitro treatment with triclabendazole. Parasitology.

[31] McConville M, Brennan GP, McCoy M, Castillo R, Hernandez-Campos A, Ibarra F (2007). Immature triclabendazole-resistant *Fasciola hepatica*: Tegumental responses to in vitro treatment with the sulphoxide metabolite of the experimental fasciolicide compound alpha. Parasitol Res.

[32] Lee YJ, Shi R, Witt SN (2013). The small molecule triclabendazole decreases the intracellular level of cyclic AMP and increases resistance to stress in *Saccharomyces cerevisiae*. PLoS One.

[33] Fairweather I, Boray JC (1999). Fasciolicides: efficacy, actions, resistance and its management. Vet J.

[34] Buchanan JF, Fairweather I, Brenna GP, Trudgett A, Hoey EM (2003). *Fasciola hepatica*: surface and internal tegumental changes induced by treatment *in vitro* with the sulphoxide metabolite of albendazole (‘Valbazen’). Parasitology.

[35] Alvarez LI, Solana HD, Mottier ML, Virkel GL, Fairweather I, Lanusse CE (2005). Altered drug influx/efflux and enhanced metabolic activity in triclabendazole-resistant liver flukes. Parasitology.

[36] Robinson MW, Lawson J, Trudgett A, Hoey EM, Fairweather I (2004). The comparative metabolism of triclabendazole sulphoxide by triclabendazole-susceptible and triclabendazole-resistant *Fasciola hepatica*. Parasitol Res.

[37] Solana H, Scarcella S, Virkel G, Ceriani C, Rodríguez J, Lanusse C (2009). Albendazole enantiomeric metabolism and binding to cytosolic proteins in the liver fluke *Fasciola hepatica*. Vet Res Commun.

[38] Mottier L, Alvarez L, Fairweather I, Lanusse C (2006). Resistance-induced changes in triclabendazole transport in *Fasciola hepatica*: ivermectin reversal effect. J Parasitol.

[39] Savage J, Meaney M, Brennan GP, Hoey E, Trudgett A, Fairweather I (2013). Increased action of triclabendazole (TCBZ) in vitro against a TCBZ-resistant isolate of *Fasciola hepatica* following its co-incubation with the P-glycoprotein inhibitor, R(+)-verapamil. Exp Parasitol.

[40] Savage J, Meaney M, Brennan GP, Hoey E, Trudgett A, Fairweather I (2013). Effect of the P-glycoprotein inhibitor, R(+)-verapamil on the drug susceptibility of a triclabendazole-resistant isolate of *Fasciola hepatica*. Vet Parasitol.

[41] Devine C, Brennan GP, Lanusse CE, Alvarez LI, Trudgett A, Hoey E (2009). Effect of the metabolic inhibitor, methimazole on the drug susceptibility of a triclabendazole-resistant isolate of *Fasciola hepatica*. Parasitology.

[42] Devine C, Brennan GP, Lanusse CE, Alvarez LI, Trudgett A, Hoey E (2011). Enhancement of triclabendazole action *in vivo* against a triclabendazole-resistant isolate of *Fasciola hepatica* by co-treatment with ketoconazole. Vet Parasitol.

[43] Devine C, Brennan GP, Lanusse CE, Alvarez LI, Trudgett A, Hoey E (2012). Potentiation of triclabendazole action in vivo against a triclabendazole-resistant isolate of *Fasciola hepatica* following its co-administration with the metabolic inhibitor, ketoconazole. Vet Parasitol.

[44] Scarcella S, Miranda-Miranda E, Cossío-Bayúgar R, Ceballos L, Fernandez V, Solana H (2012). Increase of carboxylesterase activity in *Fasciola hepatica* recovered from triclabendazole treated sheep. Mol Biochem Parasitol.

[45] Scarcella S, Lamenza P, Virkel G, Solana H (2012). Expression differential of microsomal and cytosolic glutathione-S-transferases in *Fasciola hepatica* resistant at triclabendazole. Mol Biochem Parasitol.

[46] Chemale G, Perally S, LaCourse EJ, Prescott MC, Jones LM, Ward D (2010). Comparative proteomic analysis of triclabendazole response in the liver fluke *Fasciola hepatica*. J Proteome Res.

[47] Matoušková P, Vokřál I, Lamka J, Skálová L (2016). The role of xenobiotic-metabolizing enzymes in anthelmintic deactivation and resistance in helminths. Trends Parasitol.

[48] Valentim CLL, Cioli D, Chevalier FD, Cao X, Taylor AB, Holloway SP (2013). Genetic and molecular basis of drug resistance and species-specific drug action in schistosome parasites. Science.

[49] Chevalier FD, Valentim CL, LoVerde PT, Anderson TJ (2014). Efficient linkage mapping using exome capture and extreme QTL in schistosome parasites. BMC Genomics.

[50] You H, McManus DP, Hu W, Smout MJ, Brindley PJ, Gobert GN (2013). Transcriptional responses of in vivo praziquantel exposure in schistosomes identifies a functional role for calcium signaling pathway member CamKII. PLoS Pathog.

[51] Kasinathan RS, Morgan WM, Greenberg RM (2010). *Schistosoma mansoni* express higher levels of multidrug resistance-associated protein 1 (SmMRP1) in juvenile worms and in response to praziquantel. Mol Biochem Parasitol.

[52] Hines-Kay J, Cupit PM, Sanchez MC, Rosenberg GH, Hanelt B, Cunningham C (2012). Transcriptional analysis of *Schistosoma mansoni* treated with praziquantel in vitro. Mol Biochem Parasitol.

[53] Hodgkinson J, Cwiklinski K, Beesley NJ, Paterson S, Williams DJL (2013). Identification of putative markers of triclabendazole resistance by a genome-wide analysis of genetically recombinant *Fasciola hepatica*. Parasitology.

[54] Cwiklinski K, Dalton JP, Dufresne PJ, La Course J, Williams DJ, Hodgkinson J (2015). The *Fasciola hepatica* genome: gene duplication and polymorphism reveals adaptation to the host environment and the capacity for rapid evolution. Genome Biol.

[55] McNulty SN, Tort JF, Rinaldi G, Fischer K, Rosa BA, Smircich P (2017). Genomes of *Fasciola hepatica* from the Americas reveal colonization with *Neorickettsia* endobacteria related to the agents of potomac horse and human sennetsu fevers. PLoS Genet.

[56] National Research Council (USA) (2011). Committee for the Update of the guide for the care and use of laboratory animals. Guide for the care and use of laboratory animals.

[57] R Development Core Team. R: A language and environment for statistical computing: the R Foundation for Statistical Computing, Vienna, Austria; 2016. URL: https://www.R-project.org/

[58] Huber W, Carey VJ, Gentleman R, Anders S, Carlson M, Carvalho BS (2015). Orchestrating high-throughput genomic analysis with bioconductor. Nat Methods.

[59] Lawrence M, Huber W, Pagès H, Aboyoun P, Carlson M, Gentleman R (2013). Software for computing and annotating genomic ranges. PLoS Comput Biol.

[60] Love MI, Huber W, Anders S (2014). Moderated estimation of fold change and dispersion for RNA-seq data with DESeq2. Genome Biol.

[61] Benjamini Y, Hochberg Y (1995). Controlling the false discovery rate: a practical and powerful approach to multiple testing. J R Stat Soc.

[62] Alexa A, Rahnenführer J. topGO: Enrichment analysis for Gene Ontology. R Package version 2.16.0. 2010.

[63] Howe KL, Bolt BJ, Shafie M, Kersey P, Berriman M (2017). WormBase ParaSite - a comprehensive resource for helminth genomics. Mol Biochem Parasitol.

[64] Li H, Handsaker B, Wysoker A, Fennell T, Ruan J, Homer N (2009). The sequence alignment/map format and SAMtools. Bioinformatics.

[65] McLaren W, Gil L, Hunt SE, Riat HS, Ritchie GRS, Thormann A (2016). The ensembl variant effect predictor. Genome Biol.

[66] Kanehisa M, Sato Y, Morishima K (2016). BlastKOALA and GhostKOALA: KEGG tools for functional characterization of genome and metagenome sequences. J Mol Biol.

[67] Kanehisa M, Furumichi M, Tanabe M, Sato Y, Morishima K (2017). KEGG: new perspectives on genomes, pathways, diseases and drugs. Nucleic Acids Res.

[68] Gaboriaud C, Gregory-Pauron L, Teillet F, Thielens NM, Bally I, Arlaud GJ (2011). Structure and properties of the Ca2+−binding CUB domain, a widespread ligand-recognition unit involved in major biological functions. Biochem J.

[69] Laing ST, Ivens A, Laing R, Ravikumar S, Butler V, Woods DJ (2010). Characterization of the xenobiotic response of *Caenorhabditis elegans* to the anthelmintic drug albendazole and the identification of novel drug glucoside metabolites. Biochem J.

[70] Lacey E (1988). The role of the cytoskeletal protein, tubulin, in the mode of action and mechanism of drug resistance to benzimidazoles. Int J Parasitol.

[71] Chambers E, Ryan LA, Hoey EM, Trudgett A, McFerran NV, Fairweather I (2010). Liver fluke B-tubulin isotype 2 binds albendazole and is thus a probable target of this drug. Parasitol Res.

[72] Von Samson-Himmelstjerna G, Blackhall WJ, McCarthy JS, Skuce PJ (2007). Single nucleotide polymorphism (SNP) markers for benzimidazole resistance in veterinary nematodes. Parasitology.

[73] Demeler J, Krüger N, Krücken J, von der Heyden VC, Ramünke S, Küttler U (2013). Phylogenetic characterization of β-tubulins and development of pyrosequencing assays for benzimidazole resistance in cattle nematodes. PLoS One.

[74] Ryan LA, Hoey E, Trudgett A, Fairweather I, Fuchs M, Robinson MW (2008). *Fasciola hepatica* expresses multiple alpha- and beta-tubulin isotypes. Mol Biochem Parasitol.

[75] Kwa MS, Kooyman FN, Boersema JH, Roos MH (1993). Effect of selection for benzimidazole resistance in *Haemonchus contortus* on beta-tubulin isotype 1 and isotype 2 genes. Biochem Biophys Res Commun.

[76] Kwa MSG, Veenstra JG, Roos MH (1993). Molecular characterization of β-tubulin genes present in benzimidazole-resistant populations of *Haemonchus contortus*. Mol Biochem Parasitol.

[77] Fuchs M-A, Ryan LA, Chambers EL, Moore CM, Fairweather I, Trudgett A (2013). Differential expression of liver fluke β-tubulin isotypes at selected life cycle stages. Int J Parasitol.

[78] Kotze AC, Hunt PW, Skuce P, von Samson-Himmelstjerna G, Martin RJ, Sager H (2014). Recent advances in candidate-gene and whole-genome approaches to the discovery of anthelmintic resistance markers and the description of drug/receptor interactions. Int J Parasitol Drugs Drug Resist.

[79] Messerli SM, Kasinathan RS, Morgan W, Spranger S, Greenberg RM (2009). *Schistosoma mansoni* P-glycoprotein levels increase in response to praziquantel exposure and correlate with reduced praziquantel susceptibility. Mol Biochem Parasitol.

[80] Mansour TE (1979). Chemotherapy of parasitic worms: new biochemical strategies. Science.

[81] García-Huertas P, Mejía-Jaramillo AM, González L, Triana-Chávez O (2017). Transcriptome and functional genomics reveal the participation of adenine phosphoribosyltransferase in *Trypanosoma cruzi* resistance to benznidazole. J Cell Biochem.

