# Supplementary figures and images for: Pleiotropic alterations in gene expression in Latin American Fasciola hepatica isolates with different susceptibility to drugs
Source: Parasit Vectors. 2018 Jan 24;11:56. doi: 10.1186/s13071-017-2553-2 (PMC5781333; doi:10.1186/s13071-017-2553-2)

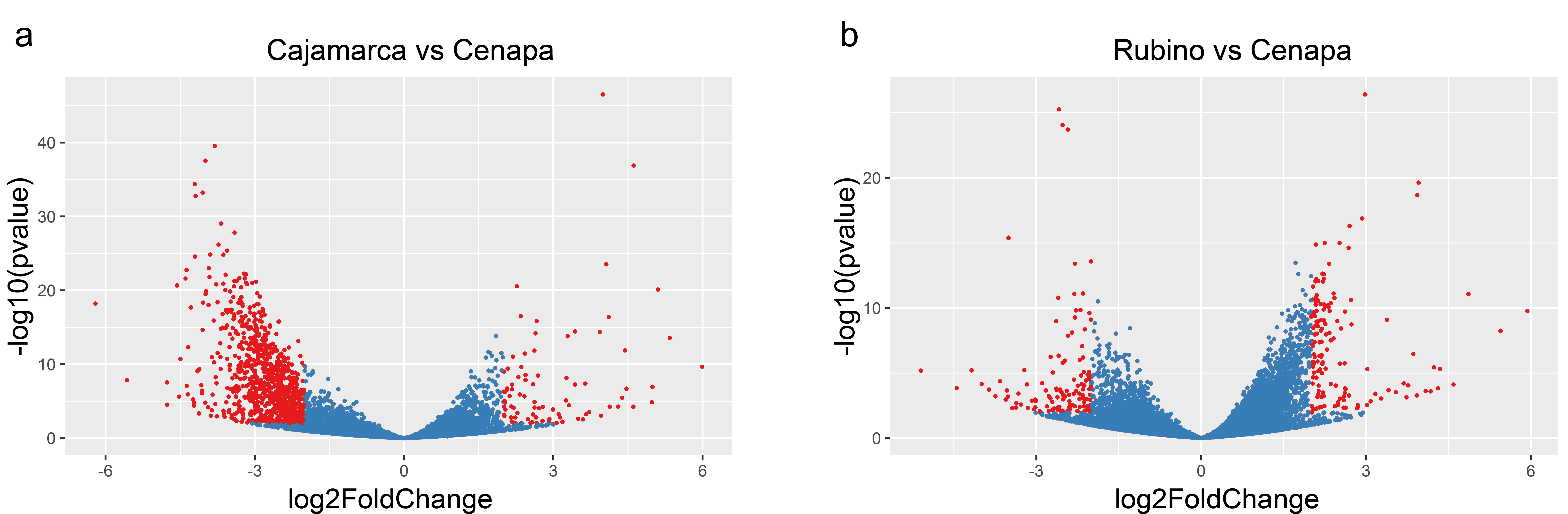

Supplement: Supplementary file 2 — Differential expression between isolate pairs. Volcano plot showing the differential expression of transcripts between Cajamarca and Cenapa (a) and between Rubino and Cenapa (b). Red dots represent differentially expressed genes (log2 fold change > 2, P-value < 0.01). (TIFF 1086 kb) [file 13071_2017_2553_MOESM2_ESM.tif]

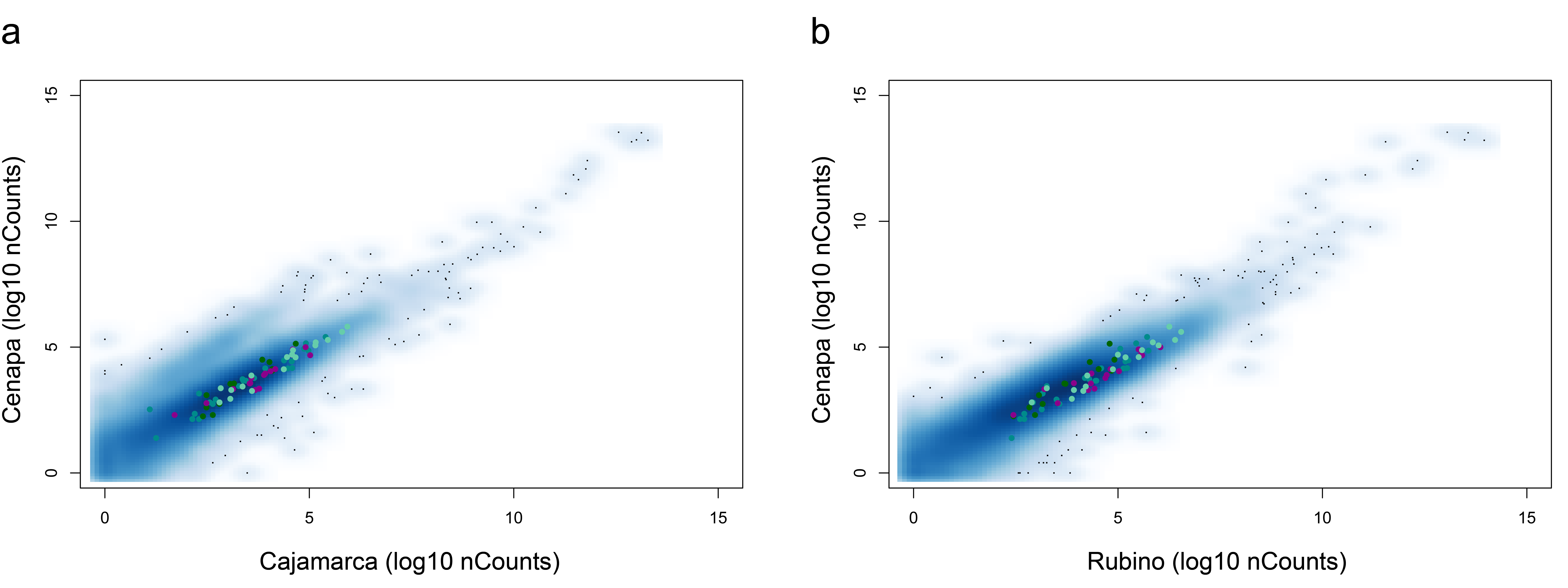

Supplement: Supplementary file 3 — a Correlation of normalized counts and housekeeping genes. Scatterplot showing the correlation of Cenapa normalized counts per gene versus their Cajamarca counterparts. b Scatterplot showing the correlation of Cenapa normalized counts per gene versus their Rubino counterparts. Full circles highlight the expression of genes belonging to housekeeping functions. Colors are as in Fig. 2. (TIFF 1042 kb) [file 13071_2017_2553_MOESM3_ESM.tif]
